# Physiological Mechanism of Waterlogging Stress on Yield of Waxy Maize at the Jointing Stage

**DOI:** 10.3390/plants12173034

**Published:** 2023-08-23

**Authors:** Xuepeng Zhang, Chao Huang, Ye Meng, Xuchen Liu, Yang Gao, Zhandong Liu, Shoutian Ma

**Affiliations:** 1 Key Laboratory of Crop Water Use and Regulation, Ministry of Agriculture and Rural Affairs, Institute of Farmland Irrigation Research, Chinese Academy of Agricultural Sciences (CAAS), Xinxiang 453002, China; 13001297977@163.com (X.Z.); 18703737321@163.com (C.H.); mengye12121@163.com (Y.M.); finaloo@163.com (X.L.); gaoyang@caas.cn (Y.G.); 2 Crop Research Institute, Shandong Academy of Agricultural Sciences, Jinan 250100, China; 3 Field Observation and Research Station of Efficient Water Use for Agriculture, Xinxiang 453002, China; 4 School of Faculty Engineering, University of Putra Malaysia, Selonga 43400, Malaysia

**Keywords:** waxy maize, waterlogging threshold, source-sink, stem lodging, potential kernel number

## Abstract

In the main agricultural area for waxy maize production in China, waterlogging occurs frequently during the waxy maize jointing stage, and this causes significant yield reduction. It is very important to understand the physiological mechanism of waterlogging stress in waxy maize during the jointing stage to develop strategies against waterlogging stress. Therefore, this study set waterlogging treatments in the field for 0, 2, 4, 6, 8, and 10 days during the waxy maize jointing stage, and were labelled CK, WS2, WS4, WS6, WS8 and WS10, respectively. By analyzing the effect of waterlogging on the source, sink, and transport of photoassimilates, the physiological mechanism of waterlogging stress in the jointing stage was clarified. The results show that PEPC and POD activities and Pro content decreased significantly under WS2 compared to CK. Except for these three indicators, the Pn, GS, leaf area, kernel number, yield, and puncture strength of stems were significantly decreased under the WS4. Under the WS6, the content of MDA began to increase significantly, while almost all other physiological indices decreased significantly. Moreover, the structure of stem epidermal cells and the vascular bundle were deformed after 6 days of waterlogging. Therefore, the threshold value of waterlogging stress occured at 4 to 6 days in the jointing stage of waxy maize. Moreover, waterlogging stress at the jointing stage mainly reduces the yield by reducing the number of kernels; specifically, the kernel number decreased by 6.7–15.5% in 4–10 days of waterlogging, resulting in a decrease of 9.9–20.2% in the final yield. Thus, we have shown that waterlogging stress at the jointing stage results in the decrease of potential waxy maize kernel numbers and yield when the synthesis of sources was limited and the transport of photoassimilates was restricted.

## 1. Introduction

Climate change is resulting in more extreme weather events, such as drought, high temperature, and soil waterlogging, etc., all significantly affecting agricultural crop production. Globally, after drought, soil waterlogging is one of the most damaging abiotic stresses [1]. In the Huang-Huai-Hai region of China, most rainfall and extreme rainfall events occurs during the summer maize (*Zea mays* L.) growing season, and the growth and yield of summer maize are significantly affected by excessive rainfall. Recent research indicates that the worst year of excessive rainfall could reduce maize yield by more than 30% in this region [2,3,4]. With the improvement and adjustment of dietary structure, the population’s demand for waxy maize is increasing. At present, China is the world’s second-largest producer of waxy maize, and the Huang-Huai-Hai region is its main producing area [5]. Compared to common maize, waxy maize has weak growth and poor stress resistance [6], so the waterlogging stress in summer will seriously affect the production of waxy maize in this region.

In contrast to the plant’s shoots, the roots are the first organs that face a decrease in oxygen tension upon waterlogging stress, and they undergo significant phenotypic changes and greater growth impairment [2,7]. Waterlogging causes soil hypoxia, reduces root activity, and affects the absorption of nutrient elements, thus, accelerating root senescence, limiting the growth and development of the root system, and disordering the growth of above-ground organs [8,9]. In the above-ground organs, waterlogging results in the closure of the leaf stomata which leads to rapid reduction in leaf transpiration rate [10]. The resulting hypoxic stress leads to the accumulation of reactive oxygen species (ROS) in plants, which damage the chloroplast membrane lipid structure and the activities of photosynthetic enzymes (e.g., ribulose-1,5-bisphosphate carboxylase (RuBPCase) and phosphoenolpyruvate carboxylase (PEPCase)) and accelerate leaf senescence [11,12,13]. Therefore, the net photosynthetic rate decreases, and the accumulation of photosynthetic matter is significantly decreased, ultimately resulting in a remarkable decline in maize yield due to waterlogging. Moreover, the stalk cortex thickness, the area of the vascular bundle and its surrounding mechanical tissue, are related to lodging resistance. Waterlogging reduces the maize stalk width, stalk rind penetration strength and bending properties, stalk cortex thickness, vascular bundle sheath thickness, and vascular bundle number, resulting in a decrease in lodging resistance [14,15,16].

Previous studies showed that waterlogging had the greatest effect on maize production from the jointing stage (V6) to the tasselling stage (VT), and most of the research has focused on common maize [4,17,18,19]. The main vegetative growth stage of maize is from V6 to VT, and stress at this stage will lead to insufficient accumulation of nutrients, thus, limiting the transport of nutrients to the grain at grain-filling stage [20]. Our previous study of soil waterlogging impacts on waxy maize also showed that waterlogging at V6-VT had the highest impact on waxy maize growth, fresh ear yield, and grain quality [21]. However, the physiological mechanism of waterlogging stress on the yield of waxy maize at V6 is still unclear. Therefore, the objectives of this study were to (1) determine the waterlogging stress threshold of waxy maize to guide management during waterlogging in production, and (2) clarify the mechanism by which waterlogging stress leads to yield reduction at V6-VT. The results can provide the necessary theoretical basis and basic data support for the research and development of new technology and new waxy maize breed varieties that can better cope with waterlogging stress.

## 2. Results

### 2.1. Yield Traits

With the extension of waterlogging time at the jointing stage, the fresh yield decreased significantly after waterlogging for more than 4 days, by 9.9–20.2% (Table 1). Furthermore, the numbers of kernels significantly decreased by 6.7–15.5% after waterlogging for more than 4 days. Waterlogging had no significant effect on kernel weight. Ear length decreased significantly under WS10, while ear diameter decreased significantly under WS4.

### 2.2. Photosynthetic Parameters

**Figure 1:**
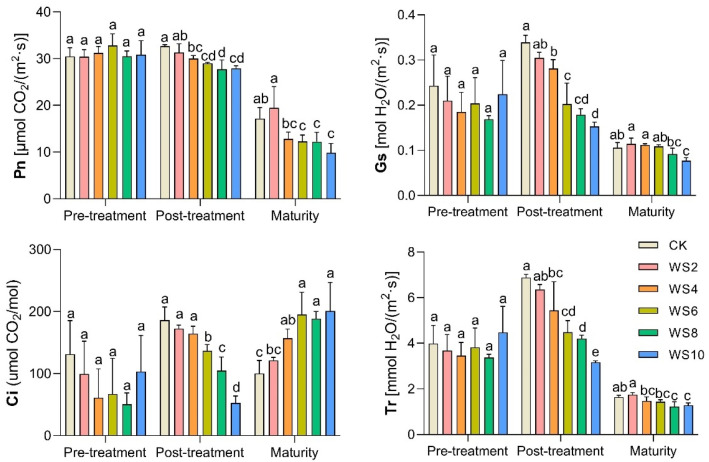
Changes of photosynthetic parameters under different waterlogging duration. Note: Different letters (a, b, c, d) above the bars indicate statistical significance (*p* ≤ 0.05).

Compared with the CK, the net photosynthetic rate (Pn) of the leaf decreased significantly with waterlogging for more than 4 days, and the Pn decreased by 8.1–15.1% (Figure 1). At the same time, the effect of waterlogging on the Pn of the leaf lasted until the maturity of waxy maize, and the Pn decreased by 25.5–42.8%, which was much larger than that at post-treatment. The stomatal conductance (Gs), intercellular CO_2_ concentration (Ci), and transpiration rate (Tr) of the post-treatment all decreased gradually with increasing waterlogging time, which is consistent with the changing trend of Pn. At the maturity stage, the trends of Pn, Gs and Tr were the same; they all decreased with longer waterlogging time. In contrast, the Ci increased significantly with longer waterlogging time.

### 2.3. Leaf Area and Phosphoenolpyruvate Carboxylase

**Figure 2:**
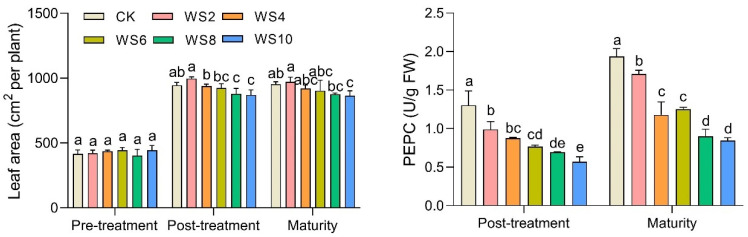
Changes of leaf area and phosphoenolpyruvate carboxylase (PEPC) activity under different waterlogging duration. Note: Different letters (a, b, c, d, e) above the bars indicate statistical significance (*p* ≤ 0.05).

With the duration of waterlogging exceeding 4 days, the leaf area per plant of maize gradually decreased significantly (Figure 2). Under the WS8 and WS10, the significantly decreasing effect on plant leaf area lasted until maize harvest. In addition, compared to the CK, the leaf area of WS8 and WS10 decreased dramatically by 8.1% and 9.4%, respectively. PEPC, which is an important restriction enzyme in the dark reaction of leaf photosynthesis, decreased markedly by 24.1–56.3% with increasing duration of waterlogging. Furthermore, the negative effects of waterlogging on PEPC activity lasted until maize maturity.

### 2.4. Plant Peroxidation Response

**Figure 3:**
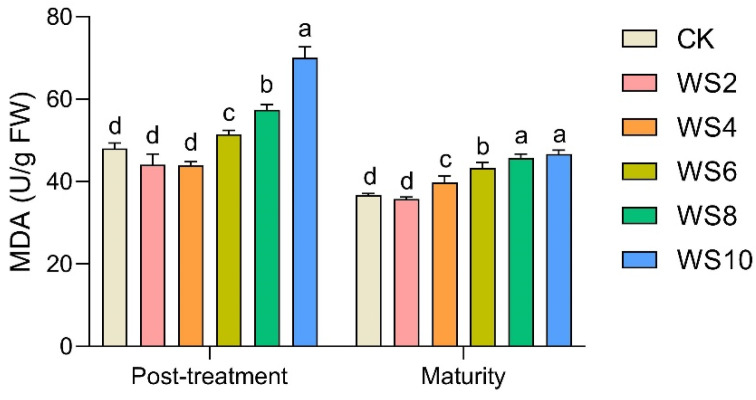
Changes of leaf Malonaldehyde (MDA) content under different waterlogging duration. Note: Different letters (a, b, c, d) above the bars indicate statistical significance (*p* ≤ 0.05).

In the post-treatment, the MDA in leaves waterlogged for more than 6 days was significantly increased by 7.3–45.8% compared to the CK (Figure 3). The negative effects of waterlogging on maize leaves still existed at maturity. Compared to the CK, the MDA significantly increased by 8.4–27.2% after more than 4 days of waterlogging.

### 2.5. Plant Antioxidant Response

**Figure 4:**
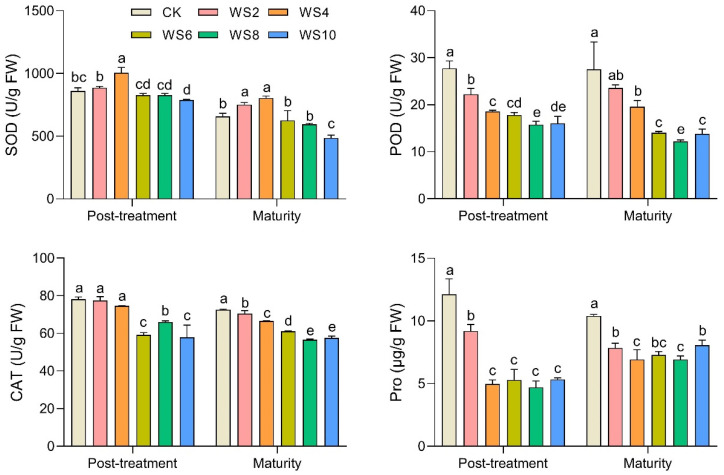
Changes of leaf antioxidant enzyme activity and proline content under different waterlogging duration. Note: SOD, superoxide dismutase; POD, peroxidase; CAT, catalase; Pro, proline; Different letters (a, b, c, d, e) above the bars indicate statistical significance (*p* ≤ 0.05).

With increasing duration of waterlogging, the SOD activity increased at first and then decreased, with the highest SOD activity in WS4 (Figure 4). However, the POD and CAT activities decreased with longer waterlogging. The POD decreased significantly after 2 days of waterlogging, while CAT decreased significantly after 6 days of waterlogging. Compared with the CK, the content of proline in leaves decreased by 24.2% in the WS2, and at more than 4 days of waterlogging, proline decreased by about 60%.

### 2.6. Evaluation of Lodging Resistance

**Figure 5:**
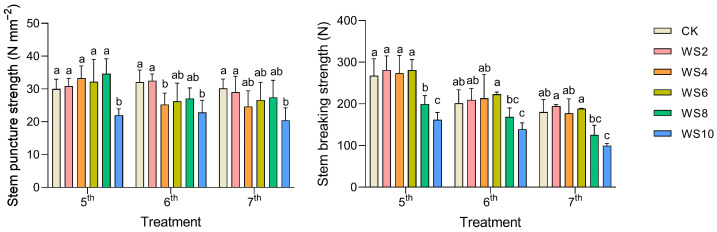
Changes of stem puncture strength and stem-breaking strength under different waterlogging duration. Note: Different letters (a, b, c) above the bars indicate statistical significance (*p* ≤ 0.05).

After 4 days of waterlogging, the stem puncture strength of the 6th internodes had decreased significantly by about 20% compared to the CK. On the 10th day of waterlogging, the stem puncture strength of the 6th and 7th internodes had decreased significantly by approximately 30% compared to the CK. However, the stem puncture strength of the 5th internodes decreased significantly only for the WS10, with a decrease of 26.6% (Figure 5).

The stem-breaking strength decreased significantly with increasing internodes. At the same time, the breaking strength of the 5th, 6th, and 7th internodes decreased significantly after 8 days of waterlogging. The stem-breaking strength of the 5th, 6th, and 7th internodes decreased by 25.4%, 16.0%, and 30.2%, respectively, compared with CK on the WS8. Under the WS10, the stem-breaking strength of the 5th, 6th, and 7th internodes significantly decreased by 39.4%, 30.9% and 44.7%, respectively, compared with CK.

### 2.7. Vascular Bundles of the Stem

**Figure 6:**
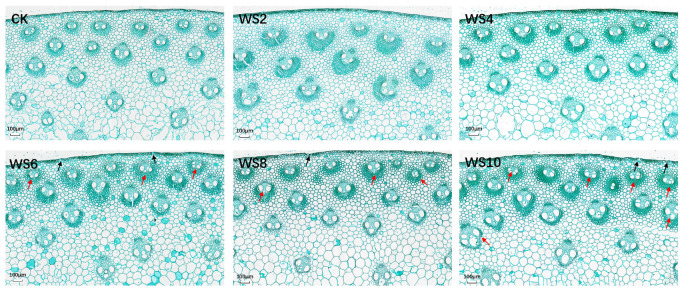
Staining of stem cross-section under different waterlogging duration. Note: The black arrows indicate the location of abnormal changes in stem epidermal cells; the red arrows indicate abnormal growth of vascular bundles.

With the increase of waterlogging treatment time, the scanning results of 6th internodes slices showed that the arrangement of stem epidermal cells with waterlogging for more than 6 days showed abnormal changes, as shown by the black arrow in Figure 6. Moreover, the number of xylems in a part of the vascular bundle increased in stems that had been waterlogged for more than 6 days, which were shown by the red arrow in Figure 6. Meanwhile, the number of abnormal changes in vascular bundle structure increased with the increase of waterlogging time.

**Figure 7:**
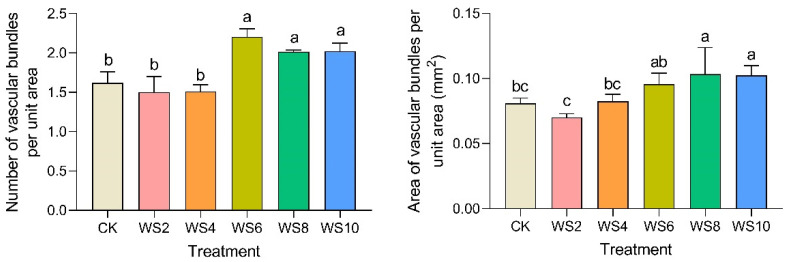
Changes of the number and area of vascular bundles under different waterlogging duration. Note: Different letters (a, b, c) above the bars indicate statistical significance (*p* ≤ 0.05).

The number and area of vascular bundles per unit area of the stem were further analyzed (Figure 7). The results showed that the number and area of vascular bundles per unit area of stem increased significantly after 6 days of waterlogging. The reason for the results observed from stem slicing may be that the number of incomplete small vascular bundles in stems increased due to waterlogging.

### 2.8. Correlation Analysis

**Figure 8:**
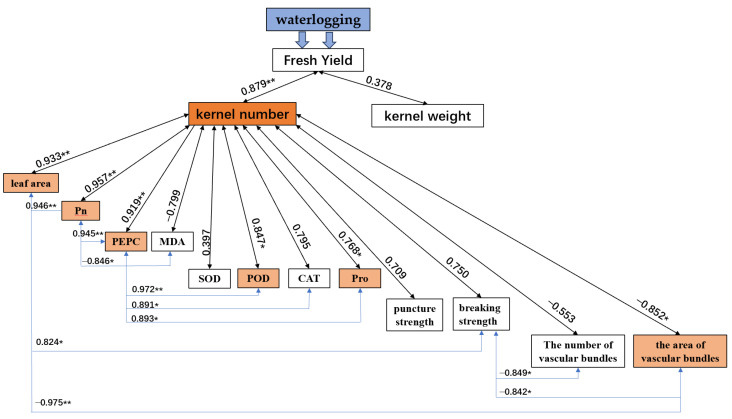
Correlation analysis between yield and physiological index. Note: ** Correlation is significant at the 0.01 level; * Correlation is significant at the 0.05 level.

The analysis of the correlations between fresh yield and yield components show that jointing stage waterlogging had no significant effect on kernel weight; however, the kernel number was significantly positively related to fresh yield (Figure 8). The leaf area, Pn, PEPC, POD, and Pro were significantly and positively related to kernel number. The area of vascular bundles was significantly and negatively related to kernel number.

The relationships between each of the physiological indices were analyzed. The Pn and breaking strength were significantly and positively related to leaf area, and the area of vascular bundles was significantly and negatively related to leaf area. The PEPC was significantly and positively related to Pn, and the MDA was significantly and negatively related to Pn. The POD, CAT, and Pro were each significantly and positively related to PEPC. The number and area of vascular bundles were significantly and negatively related to breaking strength.

## 3. Discussion

### 3.1. Effect of Waterlogging Stress on Source Synthesis

Photosynthesis is the most basic life activity of plants, and it is one of the physiological processes most sensitive to abiotic stress [22]. Research by Vandoorne et al. [23] showed that the stomata closed under waterlogging stress and gas exchange parameters declined and intercellular CO_2_ was continuously taken up. However, CO_2_ could not enter through the stomata, which resulted in an insufficient intercellular CO_2_ supply; thus, the photosynthetic rate decreased. Our results also showed that under waterlogging stress, stomata in leaves were closed which restricts gas exchange. The intercellular CO_2_ concentration (Ci) and transpiration rate (Tr) decreased, and the net photosynthetic rate (Pn) decreased due to stomatal limitation (Figure 1). These results are similar to those of Tian et al. [10].

In addition, waterlogging stress can cause the accumulation of reactive oxygen species (ROS) (e.g., OH-, O^2-^, and H_2_O_2_), membrane lipid peroxidation, and disruption of membrane homeostasis, resulting in MDA concentrations [9,21,24]. Our results showed that waterlogging significantly increased MDA content, up to 45.8% (Figure 3), and the correlation analysis also showed a significant negative correlation between MDA and Pn (Figure 8), which suggests that waxy maize had obvious membrane lipid peroxidation. The membrane lipid structure of chloroplasts and mitochondria degenerated after waterlogging [9]. Meanwhile, Ci increased significantly, while Pn decreased significantly after the end of waterlogging (Figure 1), indicating that the decrease in photosynthesis was also limited by non-stomatal factors, mainly due to the damage to chloroplast and mitochondrial membrane lipid structures and a decrease in the activity of the leaf phosphoenolpyruvate carboxylase (PEPC) after waterlogging (Figure 2). This result is similar to Tian et al. [10] and Huang et al. [21]. Furthermore, the reduction of photosynthesis means that the synthesis of maize sources is limited.

### 3.2. Responses of Antioxidant Systems under Waterlogging Stress

There are two general responses of antioxidant systems. Firstly, anoxic stress leads to ROS formation in plant cells. To neutralize the toxicity of ROS, plants have evolved an endogenous system of enzymes (e.g., CAT, POD, SOD) to operate, if exposed to stress [25]. When the production level of peroxides exceeds the scavenging capacity of antioxidant enzymes, membrane lipid peroxidation occurs and MDA levels increase [26]. The increase in ROS and MDA will induce an increase in antioxidant enzyme activity [24]. Many studies have also confirmed that waterlogging can increase SOD, POD, and CAT activities [12,13,27]. Unlike previous studies, our study showed that although the MDA increased under waterlogging, the SOD, POD, and CAT activity decreased. The reason may be that waxy maize has low waterlogging resistance. Ye et al. [11] suggest that the antioxidant enzyme (POD, SOD, and CAT) activities of waxy maize hybrids were lower, in contrast to those of normal maize. Furthermore, altered membrane properties in leaves fail to sustain turgidity and may cause osmotic stress favored by reduced water transport, which in turn presumably decreases SOD, POD, and CAT activity and leads to overproduction of ROS [12,28]. In addition, the excessive accumulation of ROS will change the pH of cells, and cause oxidative damage to SOD, POD, and CAT activity [12,28,29].

Secondly, as an osmoprotectant molecule, proline (Pro) maintains and improves the water status of plants under waterlogging stress. Proline also acts as an antioxidant, protecting cells from free radical damage and maintaining the cell environment for the better synthesis of biomolecules that play a role in stress adaptation [30]. Most studies showed that proline content increased under waterlogging stress [31,32,33]. However, the results of our study differ from those studies, but are supported by Barickman et al. [30]. There are two possible reasons: (1) the lower tolerance or higher susceptibility of waxy maize leads to reduced osmotic adjustment capacity of plant cells [33]; (2) leaf evaporation decreases when stomata are closed, and cell water content increases, which results in increased intracellular turgor. At this point, plants can increase the osmotic potential by reducing the content of Pro to facilitate the excretion of excess water from cells, thus, maintaining normal intracellular turgor.

### 3.3. Effect of Waterlogging Stress on the Transport of Photoassimilates

The transport level of photoassimilates to the sink organs is one of the important factors that determine the resistance of crops under stress. Photoassimilate supply to hypoxic roots of E. camaldulensis seedlings, a sink organ, was limited by reduced photoassimilate transport rather than by reduced photosynthesis [34]. The transport of photoassimilates is mainly through the vascular bundle structure, from the source organ to the sink organ. Waterlogging stress reduced the vascular bundle sheath thickness and the number of vascular bundles in the stem of spring maize and summer maize [14,25]. Unlike previous studies, our results show that the number and area of vascular bundles in waxy maize stems increased significantly. This may be because the number of incomplete small vascular bundles in stems increased. In addition, the number of xylems in a part of the vascular bundle increased in stems that had been waterlogged. The reason may be that the vascular bundle forms new xylem through schizogeny and lysogeny, which destroys the normal structure of the vascular bundle [12,35]. Therefore, waterlogging results in abnormal changes in the large vascular bundle of the waxy maize stems, which may be the main reason for the limitation of the transport of photoassimilates.

Stem lodging, which limits the improvement of crop yield, is a common problem in many crops [36]. Studies have indicated that, in the third internodes of spring maize and summer maize, waterlogging stress reduced the stem puncture and breaking strength, stem cortex thickness, vascular bundle sheath thickness, and vascular bundle number, which resulted in a decrease of lodging resistance [14,25,37]. Our study showed that the arrangement of stem epidermal cells was abnormally changed with prolonged waterlogging, which decreased the puncture strength and breaking strength of the waxy maize stem. Although waterlogging stress resulted in increased lodging risk, lodging plants were not detected in our study. The reason may be that the environment of the plot experiment in this study was relatively more stable than that of the field conditions.

### 3.4. Effect of Waterlogging on Waxy Maize Yield Formation

Previous studies indicated that waterlogging stress inhibited the growth and development of plants and consequently dry matter accumulation [10,19,38]. The number of ovules (potential kernels) per ear and the size of the ear is currently determined from V12 to V17 [39]. At this phase of waterlogging, due to the reduction of source synthesis and the restricted transport of photoassimilate, the leaf area and biomass decreased (Figure 2) which affected the development of ovules. This result was also confirmed by Masoni et al. [40] and Orlandi et al. [41]. Consequently, the yield decreased significantly due to the decrease in kernel number per ear (Table 1 and Figure 8). Most studies suggest that the increase of waterlogging at the jointing stage also limited grain filling and reduced maize grain weight [4,10,19]. However, the results of this study showed that there was no significant difference in kernel weight under different waterlogging times (Table 1). This may be a balancing between the components of maize yield. That is, since the source and transport of photoassimilates were also limited in the grain-filling, the kernel weight increased to a certain extent when the number of kernels per ear decreases.

### 3.5. Waxy Maize Waterlogging Threshold

We determined the waterlogging threshold of waxy maize at V6-VT by examining the significant changes of the indices under different waterlogging times. After 2 days of waterlogging (WS2), the PEPC activity, POD activity, and the Pro content decreased significantly. After 4 days of waterlogging (WS4), the Pn, GS, leaf area, kernel number, yield, and the puncture strength of the stem decreased significantly. After 6 days of waterlogging (WS6), the content of MDA increased significantly, while the SOD activity, CAT activity, and the breaking strength of the stem decreased significantly. Moreover, the structure of stem epidermal cells and vascular bundle was deformed after 6 days of waterlogging. From the above analysis, only three indices changed significantly under WS2. Under WS4, 9 indices changed significantly, including the most important yield index. Under WS6, almost all indices changed significantly. Therefore, the threshold value of waterlogging stress was 4 to 6 days at V6-VT of waxy maize. The results can be used to guide field management of waxy maize after waterlogging to minimize yield loss.

## 4. Materials and Methods

### 4.1. Site Description

The experiment was carried out in 2021 under a large-scale rain shelter at the Xinxiang Comprehensive Experimental Station of the Chinese Academy of Agricultural Sciences, which is located in Qiliying Town, Xinxiang, China (35°18′ N, 113°54′ E); the physical and chemical properties of soil are shown in Table 2. All experimental plots were made of steel sheets with irrigation and drainage systems and measured 3.33 m in length × 2.0 m in width×1.8 m in depth. The bottom layer of 20 cm of each plot was filled with mixed very coarse sands and gravels that acted as a filter layer to prevent soil loss from 150 cm of soil layer above it, while permitting normal leakage of water through. The top side of the steel outer frame of the plot is 10 cm higher than the soil surface to prevent runoff during rain or irrigation events. The upper part of the plot was equipped with a mobile rain shelter. The rain shelter was closed during rainfall and opened after rain, which can effectively control the influence of rainfall on the waterlogging test.

**Figure 9:**
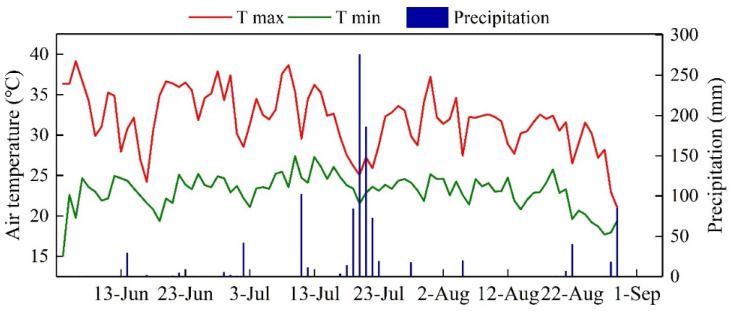
The changes in maximum daily temperature (Tmax), minimum daily temperature (Tmin) and precipitation during the whole growth period of waxy maize.

An automatic weather station was set near the edge of the experimental field. The changes in temperature and precipitation during the whole growth period of waxy maize are shown in Figure 9.

### 4.2. Experimental Design

**Figure 10:**
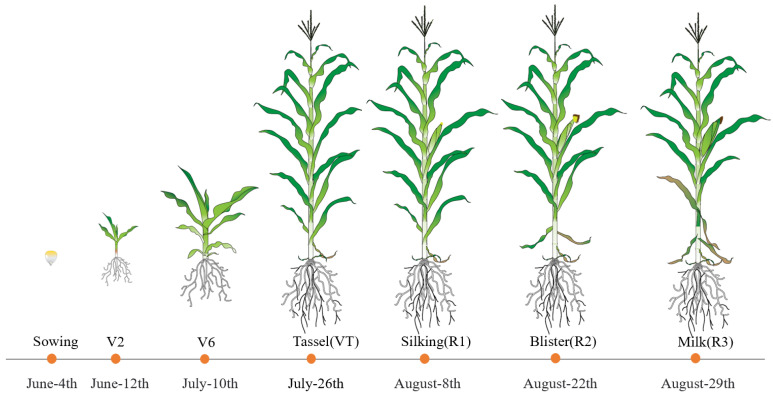
The start date of each growth stage.

The experimental variety was “Shenkenuo 602”, which was bred by the Shanghai Academy of Agricultural Sciences and widely cultivated in China. The experimental planting density was set at 60,000 plants per hectare (row spacing 60 cm, plant spacing 30 cm). The experimental field was fertilized with 750 kg ha^−1^ of compound fertilizer (containing 18% nitrogen, 10% phosphorus, and 6% potassium) during the seedbed preparation, and no fertilizer for topdressing application thereafter. Figure 10 presents the start date of each growth stage. Waterlogging was scheduled from V6, with suitable irrigation as control (CK), and each treatment had 3 replicates. Treatments were set for 2, 4, 6, 8, or 10 consecutive days of waterlogging, and the abbreviations are WS2, WS4, WS6, WS8, and WS10 respectively. The soil water content of the CK was maintained above 65%. During waterlogging, the water layer was maintained at 5~8 cm. After waterlogging, the drainage valve at the bottom of the pit was opened for drainage. The non-waterlogging stage was irrigated when soil water content reached the lower limit of 65%. The waxy maize was harvested at the milk stage (R3). Spray insecticides and avermectin at seedling and anthesis stage. Weeds were well-controlled manually.

### 4.3. Measurements

#### 4.3.1. Soil Water Content

Volumetric soil water content (VSWS, cm^−3^∙cm^−3^) in the 0–100 cm soil layer was measured once every 7 d by using the Insentek sensor (Oriental Zhigan Technology Ltd., Zhejiang, China) with a 10 cm increment. The sensor parameters were shown in [42].

#### 4.3.2. Plant Growth and Physiological, Biochemical Indexes of Maize Leaves

In each plot, three plants with the representative and similar growth status were selected and labeled at the start date of the V6 stage of waxy maize. At the pre-treatment stage (V6), post-treatment stage (VT), and maturity stage (R3), the photosynthesis was measured at 9: 00~11: 00 a.m. on sunny days by Li-6400 portable photosynthesis analyzer (LI-COR, USA). Measurement conditions were kept consistent: LED light source and the PAR was 1200 μmol/m^2^, the airflow rate was 500 µmol s^−1^, reference CO_2_ concentration was 370 µmol mol^−1^, and relative humidity was 20%. The photosynthetic parameters included net photosynthetic rate (Pn), stomatal conductance (Gs), transpiration rate (Tr), and intercellular CO_2_ concentration (Ci) [43]. On the same date, the length and maximum width of all the leaves of the three plants were measured using a ruler. The total green leaf area per plant is calculated as followed: Green leaf area (m^2^) = green leaf length × green leaf width × 0.75. Five other ear leaf samples of each treatment were taken at VT and R3. The leaves were cut into pieces and stored in liquid nitrogen for testing. The malonaldehyde (MDA), superoxide dismutase (SOD), catalase (CAT) peroxidase (POD) and phosphoenolpyruvate carboxylase (PEPC) of the samples were determined by the kit of NanJing JianCheng Bioengineering Institute (http://www.njjcbio.com (accessed on 1 December 2021)). To a weight of 0.5 g of the fresh leaf sample, 4.5 mL 0.9% normal saline was added and ground with ice water bath. Then, from samples in a refrigerated centrifuge, spun at 2500 RPM for 10 min, the supernatant was collected to be measured. The MDA, SOD, CAT, POD and PEPC were determined according to the kit instructions.

The proline (Pro) content in the sample leaf was measured by the ninhydrin method. A 0.5 g fresh leaf sample was weighed and ground with 5 mL 3% sulfosalicylic acid solution, then extracted in a boiling water bath for 10 min. After cooling, 2 mL of filtrate was absorbed and placed in a 10 mL centrifuge tube, 2 mL of ice acetic acid and 2 mL of acid ninhydrin were added. After heating in a boiling water bath for 30 min, 4 mL toluene was added for color development. Color comparison was performed at 520 nm by UV-2450 spectrophotometer. The content of proline (*X*) in 2 mL solution was calculated by standard curve. The proline content in the sample is calculated by the following formula: Pro content (μg/g) = (*X* × 5/2)/0.5.

#### 4.3.3. Evaluation of Lodging Resistance

The stem puncture strength and stem breaking strength at the 5th, 6th and 7th internodes of the stem were measured with a Digital Force Tester (YYD–1, Zhejiang Top Instrument, Hangzhou, Zhejiang, China). The sampled stem was placed on the plate of the support pillars. A 0.01 cm^2^ test probe was vertically inserted into the internode, and the displayed values of the force were recorded as the stem puncture strength. A uniform force was applied to the internode and increased steadily until the node broke. The values of the force at the moment of breaking were recorded as the stem-breaking strength [36].

#### 4.3.4. Vascular Bundles of the Stem

At maize harvest, the middle of the 6th internode of stalks was collected from each plot for paraffin sectioning. The stalk cross-sectional area was calculated as the ellipse followed by S = πab/4, in which a and b represent the major and minor axes of the ellipse, respectively. The sections were stained with Safranin-O/Fast green. White light scan was used to take photos, and CaseViewer 2.0 (3DHISTECH Ltd., Budapest, Hungary) software was used for the statistics of the number and area of vascular bundles in stalks cross-sectional.

#### 4.3.5. Fresh Grain Yield

At the late milk stage (R3) of the waxy maize, maize ears were collected with husks; 20 ears were collected from each plot and the husks removed to measure the fresh yield, ear length, ear diameter, and the number of kernel per ear. The fresh yield of maize was measured by weight. Ear length and ear diameter were measured using a ruler and vernier caliper, respectively. The number of kernels per ear was counted manually. Then, the fresh grains were manually removed, and 100 grains were randomly selected to determine the 100-grain weight.

### 4.4. Statistical Analysis

Data were analyzed using analysis of variance with Excel 2019 (Microsoft, Redmond, WA, USA). One-way ANOVA was performed using SPSS 18.0 (IBM Inc., Chicago, IL, USA), and means were compared using Duncan’s least significance difference (LSD) tests. Significance was declared at the probability level of 0.05. Figures were plotted using GraphPad Prism 9 (GraphPad Software LLC, San Diego, CA, USA).

## 5. Conclusions

During the jointing stage of waxy maize, waterlogging for 4 to 6 days could significantly reduce production. Firstly, the significant decrease in POD activity and the Pro content in plants under the WS4 led to the restriction of the removal of peroxide products. Due to the accumulation of peroxide products, the activity of PEPC, a key enzyme in photosynthesis, was reduced, thus limiting the synthesis of maize sources. Secondly, almost all physiological indexes were significantly altered under the WS6, while the larger vascular bundle structure was abnormally changed. As a result, the transport of photoassimilate was restricted. Finally, the number of ovules per ear and the size of the ears were determined in the jointing stage, and the waterlogging stress at this stage resulted in a decrease in the number of potential kernels when the synthesis of sources was limited and the transport of photoassimilate was restricted. The kernel number per ear was reduced, resulting in yield reduction.

## Figures and Tables

**Table 1:** Characters and yield of fresh ear.

Treatment	Ear Length cm	Ear Diameter mm	Kernel Number per Ear	Kernel Weight g/per Kernel	Fresh Yield t/ha
CK	18.06 ± 0.78 ab	48.30 ± 1.23 a	432.9 ± 14.12 ab	0.44 ± 0.01 a	13.04 ± 0.70 a
WS2	18.83 ± 1.02 a	48.89 ± 0.68 a	442.9 ± 16.87 a	0.44 ± 0.01 a	13.57 ± 0.36 a
WS4	17.82 ± 0.83 ab	46.50 ± 0.94 bc	385.9 ± 8.77 cd	0.43 ± 0.01 a	11.75 ± 0.14 b
WS6	17.75 ± 0.97 ab	47.69 ± 1.47 ab	403.7 ± 7.22 bc	0.43 ± 0.02 a	11.64 ± 0.96 b
WS8	17.69 ± 0.66 ab	45.50 ± 0.61 c	372.8 ± 12.85 d	0.43 ± 0.01 a	10.59 ± 0.26 d
WS10	16.78 ± 0.53 b	46.17 ± 1.35 bc	365.9 ± 21.86 d	0.42 ± 0.05 a	10.41 ± 0.52 d

Note: Different letters (a, b, c, d) within a column indicate significant differences at *p* ≤ 0.05.

**Table 2:** The physical and chemical properties of soil.

Soil Texture	pH	Soil Bulk Density	Field Capacity	Soil Organic Matter	Total Nitrogen	Available Potassium	Available Phosphorus	Available Nitrogen
		g·cm^−3^	%	g·kg^−1^	g·kg^−1^	mg·kg^−1^	mg·kg^−1^	mg·kg^−1^
Light sandy loam	8.8	1.25	24	18.85	1.09	101.02	72.01	15.61

## Data Availability

Data will be made available on request.
